# Evaluation of a Method for Determining Binaural Sensitivity to Temporal Fine Structure (TFS-AF Test) for Older Listeners With Normal and Impaired Low-Frequency Hearing

**DOI:** 10.1177/2331216517737230

**Published:** 2017-11-01

**Authors:** Christian Füllgrabe, Brian C. J. Moore

**Affiliations:** 1 Medical Research Council Institute of Hearing Research, School of Medicine, The 6123 University of Nottingham, UK; 2 Department of Psychology, University of Cambridge, UK

**Keywords:** interaural phase, suprathreshold processing, practice effects, clinical screen, sound localization

## Abstract

The ability to process binaural temporal fine structure (TFS) information was assessed using the TFS-AF test (where AF stands for adaptive frequency) for 26 listeners aged 60 years or more with normal or elevated low-frequency audiometric thresholds. The test estimates the highest frequency at which a fixed interaural phase difference (IPD) of ϕ (varied here between 30° and 180°) can be discriminated from an IPD of 0°, with higher thresholds indicating better performance. A sensation level of 30 dB was used. All listeners were able to perform the task reliably, giving thresholds well above the lowest allowed frequency of 30 Hz. The duration of a run averaged 5 min. Repeated testing of the normal-hearing listeners showed no significant practice effects. Thresholds varied markedly across listeners, but their ranking was fairly consistent across values of ϕ. Thresholds decreased (worsened) with decreasing ϕ and were lower than for a group of young listeners tested in an earlier study. There were weak to moderate, negative correlations between TFS-AF thresholds and audiometric thresholds at low frequencies (125–1000 Hz) but not at high frequencies (4000–8000 Hz). In conclusion, the TFS-AF test yielded a graded measure of binaural TFS sensitivity for all listeners. This contrasts with the TFS-LF (low-frequency) test, which measures the smallest detectable shift in IPD for a fixed frequency. The absence of practice effects and a reasonably short administration time make the TFS-AF test a good candidate for the assessment of sensitivity to changes in binaural TFS for older listeners without or with hearing loss.

## Introduction

In the cochlea, complex broadband signals such as speech are decomposed by the filtering on the basilar membrane (BM) into a series of narrowband signals. The waveform at each place on the BM can be considered as an envelope (ENV) superimposed on a more rapidly oscillating carrier, the temporal fine structure (TFS). One can distinguish between the physical ENV and TFS of the input signal (ENV_p_ and TFS_p_), the ENV and TFS at a given place on the BM (ENV_BM_ and TFS_BM_), and the neural representation of ENV and TFS (ENV_n_ and TFS_n_; Moore, 2014). In what follows, we use “TFS” as a generic term to refer to the internal representation of TFS, that is, both TFS_BM_ and TFS_n_. Sensitivity to changes in TFS does not seem essential for speech intelligibility in quiet (e.g., Van Tasell, Soli, Kirby, & Widin, 1987; Wilson et al., 1991), but it may improve speech intelligibility in the presence of interfering background sounds, presumably through enhanced perceptual segregation of the target and background (Stone, Moore, & Füllgrabe, 2011), based on differences in perceived direction (Füllgrabe & Moore, 2012, 2014; Neher, Lunner, Hopkins, & Moore, 2012; Rayleigh, 1907) and fundamental frequency (Brokx & Nooteboom, 1982; Jackson & Moore, 2013). Consistent with this, sensitivity to changes in TFS has been shown to be correlated with (a) the variability in speech-in-noise identification performance observed for young normal-hearing (YNH; Oberfeld & Klöckner-Nowotny, 2016; Ruggles, Bharadwaj, & Shinn-Cunningham, 2011) and older normal-hearing (ONH; Füllgrabe, Moore, & Stone, 2015) listeners; (b) the age-related decline in speech-in-noise identification observed for audiometrically normal-hearing listeners (Füllgrabe et al., 2015), (c) the speech-identification difficulties of unaided (Strelcyk & Dau, 2009) and aided (Hopkins & Moore, 2011; Lopez-Poveda, Johannesen, Perez-Gonzalez, Blanco, Kalluri, & Edwards, 2017) hearing-impaired (HI) listeners, and (d) self-reported hearing-aid benefit for older HI (OHI) listeners (Perez, McCormack, & Edmonds, 2014).

Given the important role of TFS information for speech perception in noisy listening environments, there has been keen interest in the development of reliable and fast tests that could be used to assess TFS sensitivity in the clinic and in large-scale research studies (Bernstein et al., 2013; Hopkins & Moore, 2010; Moore & Sęk, 2009; Sheft, Risley, & Shafiro, 2012). One such test is the TFS-LF (low-frequency) test, developed by Hopkins and Moore (2010), in which the task is to distinguish an interaural phase difference (IPD) of ϕ from an IPD of 0°, in bursts of pure tones with a fixed frequency. A two-interval, two-alternative forced choice (2I, 2AFC) task is used, with four successive tones in each interval. In one interval, selected randomly, the four tones all have the same IPD of 0°. In the other interval, the IPD alternates between 0° and ϕ in successive tones. The task is to indicate the interval in which the tones appear to be more diffuse or to move within the head. This task structure seems to make the task easy to learn, and practice effects are small or absent (Hopkins & Moore, 2010). The initial value of ϕ is usually set to 180°, and ϕ is adaptively varied to determine the threshold. Although this test has frequently been used to assess sensitivity to binaural TFS (Ernst & Moore, 2012; Füllgrabe, 2013; Füllgrabe et al., 2015; Füllgrabe, Harland, Sęk, & Moore, 2017; Hopkins & Moore, 2011; Léger, Moore, & Lorenzi, 2012; Lőcsei et al., 2016; Moore, Glasberg, Stoev, Füllgrabe, & Hopkins, 2012a; Moore, Vickers, & Mehta, 2012b; Neher et al., 2012; Oberfeld & Klöckner-Nowotny, 2016; Perez et al., 2014; Rönnberg et al., 2016; Sharma, Dhamani, Leung, & Carlile, 2014; Whitmer, Seeber, & Akeroyd, 2014), it has been repeatedly observed that some listeners are unable to perform the task, and hence no graded measure of sensitivity to TFS is obtained for those listeners.

To attempt to overcome this limitation, Füllgrabe et al. (2017) modified the TFS-LF test. In their method, referred to as the TFS-AF test (where AF stands for adaptive frequency), the IPD is fixed and the frequency of the tone is adaptively varied. It was reasoned that the ability to detect a change in IPD worsens markedly above a given frequency (Brughera, Dunai, & Hartmann, 2013; Hughes, 1940) and that this threshold frequency varies across listeners depending on their binaural TFS sensitivity. A similar rationale and method has been used in several earlier studies (Grose & Mamo, 2010; Neher, Laugesen, Jensen, & Kragelund, 2011; Ross, Fujioka, Tremblay, & Picton, 2007; Santurette & Dau, 2012; Thorup et al., 2016). To retain the desirable properties of the TFS-LF test of no or minimal practice effects, little dependence on test level, and reasonably short test duration, the structure of the TFS-AF test was made similar to that for the TFS-LF test.

In a series of experiments with YNH listeners (with audiometric thresholds ≤ 20 dB hearing level [HL] from 125 to 8000 Hz), Füllgrabe et al. (2017) established that all listeners were able to perform the TFS-AF test reliably, there was no trend for performance to improve with practice, and thresholds were strongly negatively correlated with those measured using the TFS-LF test (high frequencies at threshold for the TFS-AF test are associated with good TFS sensitivity, which in turn is associated with low thresholds in degrees for the TFS-LF test). Füllgrabe et al. (2017) also demonstrated that middle-aged and older listeners with normal audiometric thresholds below 2000 Hz, selected because they were unable to complete the TFS-LF test in previous studies, yielded a graded measure of binaural TFS sensitivity using the TFS-AF test. In addition, Moore and Sek (2016a) showed that six older listeners (aged 56, 76, 78, 84, 86, and 87 years) with mild-to-moderate hearing loss at low frequencies (and greater losses at high frequencies), who were unable to complete the TFS-LF test, were able to complete the TFS-AF test. Based on their findings, Füllgrabe et al. (2017) suggested that the TFS-AF test might be a suitable test for the assessment of the sensitivity to binaural TFS for a wide range of listeners both in the clinic and in large-scale research studies.

In the present study, we aimed (a) to establish whether there are practice effects on the TFS-AF test for ONH listeners (with audiometric thresholds ≤ 20 dB HL from 125 to 4000 Hz); (b) to determine the effect of varying the fixed IPD for these ONH listeners; (c) to extend the results of Moore and Sek (2016b) by assessing the suitability of the TFS-AF test for listeners with mild-to-moderate low-frequency hearing loss; and (d) to establish whether low-frequency hearing loss is associated with reduced performance on the TFS-AF test for older listeners. Most previous studies have not revealed a significant relationship between sensitivity to changes in IPD and audiometric thresholds (Füllgrabe, 2013; Füllgrabe et al., 2015; Hopkins & Moore, 2011; Moore et al., 2012a, 2012b; Thorup et al., 2016), although both King, Hopkins, and Plack (2014) and Moore and Sek (2016b) found weak-to-moderate but significant correlations between measures of IPD sensitivity and audiometric thresholds at low frequencies.

## General Method

This study was approved by the Cambridge Research Ethics Committee and the University of Nottingham’s School of Psychology Ethics Committee. Prior to participation, listeners provided informed written consent. They were paid an hourly wage for their services.

All listeners completed the Mini Mental State Examination (Folstein, Folstein, & McHugh, 1975) to screen for gross cognitive impairment, generally taken as indexed by scores < 24/30 points, and scored at least 28 points.

Audiometric thresholds were assessed following the procedure recommended by the British Society of Audiology (2004) and using standard calibrated equipment (audiometer, headphones, and bone conductor). Audiometric thresholds were measured for each ear for octave audiometric frequencies from 125 to 8000 Hz, as well as at 750, 1500, 3000, and 6000 Hz.

### Stimuli and Procedure for the TFS-AF Test

The ability to detect changes in IPD of low-frequency sinusoidal tones was assessed using the TFS-AF test and, in some cases, the TFS-LF test (Hopkins & Moore, 2010). For both tests, a 2I, 2AFC procedure with feedback was used. On each trial, two consecutive intervals were presented, separated by 500 ms. Each interval contained four consecutive 400-ms tones (including 20-ms raised-cosine rise/fall ramps), separated by 100 ms. In one interval, selected at random, the IPD of all tones was 0° (the standard). In the other interval (the target), the first and third tones were the same as in the standard interval while the second and fourth tones differed in their IPD by ϕ. Listeners with “normal” TFS sensitivity perceive pure tones with IPD = 0° as being close to the center of the head, while tones with a sufficiently large IPD are perceived as being lateralized toward one ear or the other, or as being more diffuse. Listeners were asked to indicate which of the two intervals contained a sequence of tones that appeared to change in some way, for example, to move within the head. The intervals were clearly indicated by boxes on the screen labeled “1” and “2,” which were lit during the corresponding interval. Feedback was provided after each trial by the words “correct” or “incorrect” on the screen and by “flashing” the box for the selected interval with a green light (for correct) or a red light (for incorrect).

For the TFS-AF test, the frequency of the tones was adaptively adjusted, using a 2-up, 1-down stepping rule to estimate the 71% correct point on the psychometric function (Levitt, 1971). For the TFS-LF test, the difference in IPD was changed using a 2-down, 1-up rule. For the TFS-AF test, the frequency was changed by a factor of 1.4 until the first reversal, then by a factor of 1.2 until the next reversal, and by a factor of 1.1 thereafter. The corresponding factors for the TFS-LF test were 1.95, 1.56, and 1.25. After eight reversals, the run was terminated and the geometric mean of the values of the manipulated variable at the last six reversals was taken as the threshold estimate. For the TFS-AF test, the lowest allowed frequency was 30 Hz. If the adaptive procedure called for a value below 30 Hz, the frequency was set to 30 Hz.

Because the adaptive procedures involved multiplying the frequency (or the phase) by certain factors, all threshold estimates were based on geometric means, and all statistical analyses were based on the log-transformed thresholds.

The level of presentation in each ear for each test frequency was individually adjusted to approximately 30 dB sensation level based on the measured audiometric thresholds. This allows testing of listeners with audiometric thresholds up to about 60 dB HL without loudness discomfort, unless the listener has unusually low loudness discomfort levels. The audiometric thresholds were converted to thresholds in dB sound pressure level using values of the monaural minimum audible pressure estimated from the loudness model of Moore and Glasberg (2007), and then the level was set 30 dB higher than the threshold in dB sound pressure level, based on the known sensitivity of the headphones. It was assumed that the headphones had a reasonably flat response at the eardrum over the relevant frequency range. This was the case for the Sennheiser HDA200 headphones used; the response was within ±2.6 dB from 125 to 2000 Hz, as measured using KEMAR (Burkhard & Sachs, 1975). For the TFS-AF test, the required levels at intermediate frequencies were estimated by linear interpolation (in dB on a logarithmic frequency scale), or, in rare cases (when the frequency was below 125 Hz) by extrapolation. The starting values of the tracking variables for the TFS-AF and the TFS-LF tests were 200 Hz and 180°, respectively. The fixed frequency for the TFS-LF test was 250 Hz. For a few listeners who reported that they could not hear a difference between the two intervals at the start of a TFS-AF run, the run was stopped, and the starting frequency was set to 100 Hz. Further details of the TFS-AF test can be found in Füllgrabe et al. (2017).

Stimuli were digitally synthesized using a PC and were converted to analog form using an external RME babyface soundcard with 24-bit resolution and a sampling rate of 48000 Hz. Stimuli were presented via Sennheiser HDA200 headphones. Listeners were seated in a double-walled sound-attenuating booth and chose to enter their responses via either mouse clicks on virtual buttons displayed on a monitor or manual presses of buttons on a response box.

## Experiment 1: The Effect of Practice on the TFS-AF Test for ONH Listeners

### Rationale and Method

For YNH listeners, the ability to detect changes in IPD does not change with prolonged practice for the TFS-LF test (Hopkins & Moore, 2010) or for the TFS-AF test (Füllgrabe et al., 2017). In this experiment, we assessed the effects of practice on the TFS-AF test for ONH listeners, who initially might show more procedural (i.e., task) learning, due to their lesser experience with (e.g., Czaja & Sharit, 1993) and possible negative attitude and anxiety toward (e.g., Zygouris & Tsolaki, 2015) computerized testing. The value of ϕ was 180°. All ONH listeners completed 15 threshold runs distributed over three test sessions, scheduled on different days. During the first session, three threshold runs were obtained without providing any prior practice runs. This was done to mimic roughly the test conditions and time constraints found during an audiological assessment or during research studies. However, to familiarize the listeners with the test stimuli, some example four-tone sequences containing an IPD of 0° or 180° were presented. During each of the remaining two sessions, separated by not more than five days, six threshold runs were obtained with short breaks between test blocks of three threshold runs.

### Listeners

Thirteen ONH listeners (seven females) aged from 63 to 83 years (*M* = 71.5 years; standard deviation, *SD* = 5.9) were tested. All listeners bar one had audiometric thresholds in each ear ≤ 20 dB HL between 125 and 4000 Hz; the oldest ONH listener (ONH_13_ aged 83 years) had higher thresholds (of 25 to 40 dB HL) at 3000 and 4000 Hz but was included in the study to cover a wider age range and because the elevated thresholds were outside the range of frequencies tested here. Interaural differences in audiometric threshold were ≤ 10 dB for all frequencies below 3000 Hz. Individual and mean audiometric thresholds are given in the upper part of Table 1.

**Table 1.** Age (Years), Gender (F = Female; M = Male), and Audiometric Thresholds (dB HL) for the Left and Right Ears of the Older Normal-Hearing (ONH) and Older Hearing-Impaired (OHI) Listeners.

Listener	Age	Gender	Frequency (Hz), Left ear/Right ear										
			125	250	500	750	1000	1500	2000	3000	4000	6000	8000
ONH_1_	63	F	10/10	5/5	5/5	5/5	5/5	5/5	5/10	5/10	5/10	15/15	25/30
ONH_2_	64	F	15/10	10/10	5/10	5/5	20/10	20/20	20/20	5/15	20/15	20/15	30/30
ONH_3_	67	M	15/10	10/5	0/5	5/5	5/0	5/5	5/10	10/15	5/10	20/30/	20/25
ONH_4_	67	F	10/10	5/10	5/0	5/5	5/15	5/10	10/10	0/20	5/15	15/20	45/55
ONH_5_	67	F	15/15	10/10	5/10	5/10	5/15	5/10	10/10	20/15	15/0	20/10	40/25
ONH_6_	68	F	10/10	10/5	10/10	10/10	10/10	5/5	10/5	10/15	20/20	30/30	60/55
ONH_7_	72	M	5/10	5/5	−5/−5	5/5	0/10	5/5	5/5	15/15	20/15	30/35	65/70
ONH_8_	72	M	5/10	0/5	0/5	5/15	5/5	5/10	5/10	10/10	15/10	35/10	20/20
ONH_9_	74	F	10/10	10/5	5/5	5/10	10/5	10/15	20/10	20/20	20/10	35/15	55/25
ONH_10_	74	M	15/15	10/10	0/0	10/5	10/20	5/15	5/5	20/5	20/20	35/30	45/50
ONH_11_	75	M	10/15	15/10	0/5	10/0	5/0	10/10	10/15	10/10	15/15	25/15	30/25
ONH_12_	79	F	20/20	20/20	15/20	10/10	20/15	10/15	20/15	15/10	15/15	25/30	55/50
ONH_13_	83	M	15/10	10/10	0/5	5/5	5/0	5/10	10/15	25/30	25/40	60/35	60/70
**ONH_Mean_**	**71**	**7F/6M**	**12/12**	**9/8**	**3/6**	**7/7**	**8/8**	**7/10**	**10/11**	**13/15**	**15/15**	**28/22**	**42/41**
OHI_1_	68	M	20/35	25/35	35/30	40/40	45/45	50/60	45/55	45/55	50/60	50/60	50/30
OHI_2_	69	M	25/20	35/25	40/30	45/45	55/45	55/45	55/45	55/55	55/50	65/50	75/65
OHI_3_	69	M	30/30	30/30	25/25	25/20	30/30	35/45	35/45	55/55	60/60	50/65	80/85
OHI_4_	73	M	15/15	20/20	35/35	40/40	45/45	45/55	60/50	65/60	70/70	65/70	70/65
OHI_5_	74	M	30/20	30/25	25/30	30/35	30/35	30/35	35/35	50/40	60/50	70/75	70/75
OHI_6_	77	M	20/15	40/40	45/40	40/35	55/40	60/55	60/55	60/60	60/60	65/65	85/75
OHI_7_	80	F	40/50	50/55	50/45	50/50	50/50	50/50	50/50	45/50	55/45	55/60	55/70
OHI_8_	80	M	15/25	20/35	45/45	45/40	40/35	35/40	35/40	40/40	50/50	50/55	55/60
OHI_9_	81	F	50/55	50/55	50/50	45/50	50/55	55/55	50/55	50/55	55/60	60/60	85/80
OHI_10_	81	M	35/50	35/50	35/45	45/45	50/50	55/60	60/55	55/65	50/60	75/80	85/95
OHI_11_	83	F	55/40	45/35	35/30	35/25	30/25	25/35	35/40	45/45	50/50	65/70	85/85
OHI_12_	84	F	10/20	20/25	30/35	35/35	35/40	45/40	50/45	45/50	50/45	55/50	75/75
OHI_13_	85	F	15/15	25/25	35/40	30/35	30/35	35/35	45/40	55/40	65/50	85/70	90/85
**OHI_Mean_**	** 77**	** 5F/8M**	** 28/30**	** 33/35**	** 37/37**	** 39/38**	** 42/41**	** 44/47**	** 47/47**	** 51/51**	** 56/55**	** 62/64**	** 74/73**

*Note*. HL = hearing level.

### Results

A run was considered as valid if the *SD* of the log values of the last six reversals was ≤ 0.2. All 13 listeners gave valid threshold estimates for all 15 runs. All threshold values were well above the lowest allowed frequency of 30 Hz.

The time taken to complete a threshold run ranged from 2 min 47 s to 10 min 25 s. This relatively large range was mainly due to four outliers, with run durations in excess of 9 min. After removing those values, the mean run duration was 5 min 7 s, similar to that for YNH listeners (Füllgrabe et al., 2017). Run durations excluding the four outliers ranged from 2 min 47 s to 7 min 19 s, and 90% of the runs took between 3 min 27 s and 6 min 59 s. The variability arose mainly from the variation of the number of trials within a run, even when the effect of the age of the listener was partialled out (as confirmed by a partial Pearson correlation between run duration and number of trials of .964; *p* ≤ .001; two-tailed). Longer trials were associated with a large frequency separation between the starting frequency and the threshold value, and with large ratios between successive reversal points.

**Figure 1.**
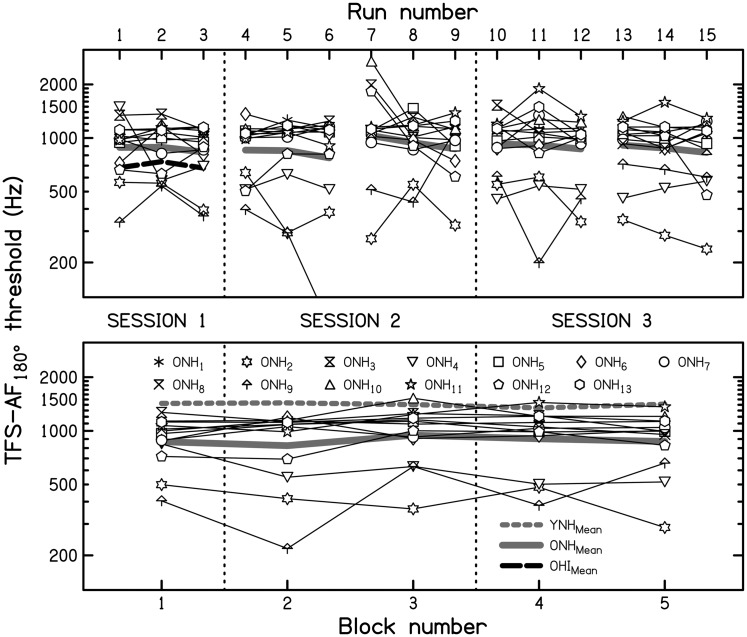
Results of Experiment 1 showing individual (thin lines) and mean (continuous thick gray line) thresholds for the TFS-AF test, using ϕ = 180°. The frequency at threshold is plotted for 13 older normal-hearing (ONH) listeners for 15 consecutive threshold runs (top panel) distributed over 3 test sessions. Threshold runs were conducted in blocks of three, separated by breaks. Individual (thin lines) and geometric mean (continuous thick gray line) thresholds for each of the five test blocks are shown in the bottom panel. The dashed thick gray line indicates geometric mean results for nine young normal-hearing (YNH) listeners from Füllgrabe et al. (2017) tested under identical conditions to those used here. The dashed thick black line in the top panel shows mean results for the three runs for the older hearing-impaired (OHI) listeners tested in Experiment 3. TFS-AF = temporal fine structure-adaptive frequency.

The upper panel of Figure 1 shows the individual thresholds (thin lines) and the geometric means across listeners (continuous thick gray line) for each run number. The lower panel shows the geometric mean across blocks of three runs for each listener (thin lines) and the geometric mean across listeners (continuous thick gray line). For comparison, mean results for a group of nine YNH listeners over the same practice period, taken from Füllgrabe et al. (2017), are shown by the dashed thick gray line. The grand geometric mean across all runs was 892 Hz, 490 Hz lower (worse) than the grand geometric mean for the YNH listeners (Füllgrabe et al., 2017). Individual thresholds and thresholds blocked across three runs varied markedly across listeners from 238 to 2698 Hz and 287 to 1525 Hz, respectively. Thresholds as high as 2698 Hz probably resulted from several lucky guesses, because such values are well above the highest frequency for which changes in IPD are detectable even for young listeners with normal hearing, which is about 1500 Hz (Brughera et al., 2013; Hughes, 1940). Thresholds above 1500 Hz occurred for 6 individual runs out of 195 (i.e., 3%).

The variability in binaural TFS sensitivity across the ONH listeners did not depend on the age of the listeners, as average thresholds across all runs were not significantly correlated with age; Spearman’s *ρ* = .252, *p* = .406; two-tailed (here and in the remainder of this article, nonparametric tests were used when the data violated the assumptions of normality or homogeneity of variance). Some previous studies have shown that binaural TFS sensitivity worsens with increasing age for normal-hearing listeners (Füllgrabe, 2013; Moore et al., 2012b; Ross et al., 2007), but these studies used listeners with a greater age range than employed here. A reanalysis of the data of Füllgrabe et al. (2015) for 21 ONH listeners aged 60 to 79 years also showed no significant correlation between age and TFS-LF thresholds at 500 Hz (Spearman’s *ρ* = −.110, *p* = .634; two-tailed) and 750 Hz (Spearman’s *ρ* = −.293, *p* = .197; two-tailed). However, this finding and the results from the current study are at odds with the observation of Moore et al. (2012a) that binaural TFS sensitivity was significantly correlated with age for 39 listeners, aged 61 to 83 years with normal or near-normal low-frequency audiometric thresholds.

Consistent with findings from previous studies with YNH listeners performing the TFS-LF (Hopkins & Moore, 2010) or TFS-AF test (Füllgrabe et al., 2017), there were no noticeable effects of practice for our ONH listeners, either in terms of improvements or in terms of deterioration due to fatigue, boredom, or loss of motivation. A Friedman’s analysis of variance (ANOVA) was conducted with the threshold for each block as the variable (data in the lower panel of Figure 1) and yielded a χ^2^_(4)_ of 4.369, which was not significant (*p* = .358). In addition, linear regression analyses (threshold vs. block number) for each listener and the entire group showed that none of the slopes was significantly different from zero (all *p* ≥ .064 without correction for multiple comparisons; two-tailed).

## Experiment 2: The Effect of the Value of ϕ for ONH Listeners

### Rationale and Method

In the previous experiment, the value of ϕ was fixed at 180°, corresponding to the maximum IPD possible. This was done to ensure that clear differences between the two intervals were heard for as many listeners as possible. However, for this antiphase condition, the side of lateralization is ambiguous, with sounds being heard sometimes to the left and sometimes to the right. In addition, as shown in the first experiment, the threshold for the TFS-AF test was lower for ONH listeners than for YNH listeners. For low frequencies (below about 770 Hz), a value of ϕ of 180° leads to interaural time differences larger than would naturally occur, and to which listeners might be less sensitive (Kunov & Abel, 1981; Mossop & Culling, 1998). The purpose of the second experiment was to investigate the effect of the value of ϕ on TFS-AF thresholds, using values of 30, 45, 90, and 135°.

Only 12 of the original 13 ONH listeners were tested, as listener ONH_9_ was no longer available. As all listeners had completed 15 runs of the TFS-AF test, no further practice was given prior to administration of three test blocks, each composed of one threshold run for each of the four values of ϕ, presented in random order. The final estimate of TFS-AF threshold was based on the geometric mean of the three valid threshold runs for a given value of ϕ. When the *SD* of the log-transformed threshold values across the three runs exceeded 0.20, an additional run was conducted, and the final estimate was taken as the geometric mean of all four estimates. Füllgrabe et al. (2017) showed that IPD sensitivity for YNH listeners, as measured by the TFS-AF test, declined abruptly when the value of ϕ divided by the IPD threshold at 250 Hz, assessed using the TFS-LF test, was less than about 3 (this ratio is denoted R). To assess whether this was also the case for the ONH listeners, two threshold runs for 500-ms, 250-Hz pure tones presented at 30 dB HL were obtained using the TFS-LF test (for further details, see Füllgrabe et al., 2015; Hopkins & Moore, 2010).

### Results

**Figure 2.**
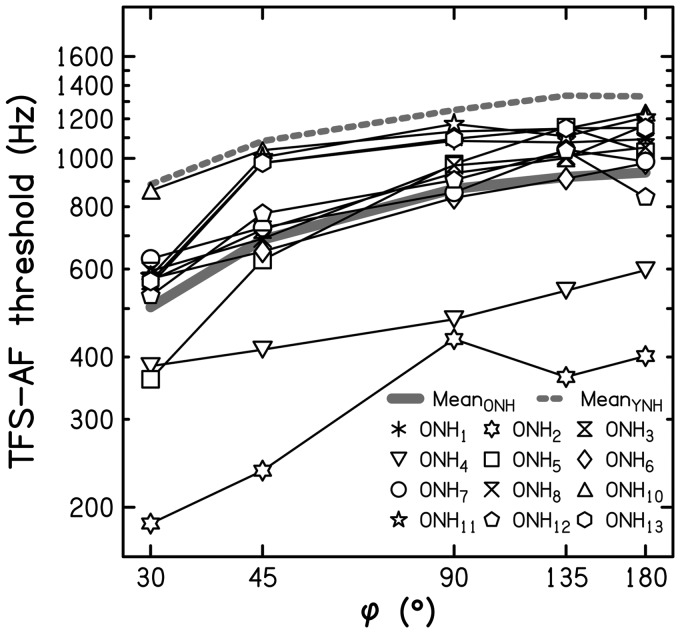
Mean individual thresholds (thin lines) and overall mean thresholds (continuous thick gray line) for the TFS-AF test as a function of ϕ for 12 older normal-hearing (ONH) listeners. The dashed thick gray line indicates mean thresholds for the nine YNH listeners from Füllgrabe et al. (2017). TFS-AF = temporal fine structure-adaptive frequency; YNH =young normal-hearing.

Individual (thin lines) and geometric mean (continuous thick gray line) TFS-AF thresholds for the ONH listeners are plotted in Figure 2 as a function of ϕ. The individual differences found in Experiment 1 using ϕ = 180° (and replotted in Figure 2) persisted across the lower values of ϕ tested here. For example, ONH_2_ performed consistently poorly, and ONH_10_ performed consistently well. Spearman correlation coefficients between each listener’s thresholds obtained with ϕ = 180° and each of the other values of ϕ ranged from .615 to .902 (all *p* ≤ .033, two-tailed and without correction for multiple comparisons). Thresholds varied only slightly for values of ϕ from 90° to 180° but declined markedly for values of ϕ below 90°. A Friedman’s ANOVA was conducted to assess the significance of differences between thresholds for the different values of ϕ. This yielded a χ^2^_(4)_ of 40.07, which was significant (*p* < .001). Subsequent Wilcoxon signed-rank tests (uncorrected for multiple comparisons) between thresholds for the maximum value of ϕ and for each smaller value of ϕ confirmed that there was no significant difference for the two largest values of ϕ (135° and 180°; *z* = −0.628, *p* = .530; 2-tailed), but there was a significant decline for the three smallest values of ϕ relative to the maximum value (all *z* ≤ −2.040, all *p* ≤ .041; 2-tailed).

For comparison, geometric-mean results for the nine YNH listeners from Füllgrabe et al. (2017) are indicated by the dashed thick gray line. The difference in mean threshold for the two age groups (YNH vs. ONH) was fairly stable (ranging from a minimum of 381 Hz to a maximum of 418 Hz for values of ϕ of 90° and 135°, respectively) and, on average, 394 Hz. The corresponding ratios (YNH/ONH) of 1.8 for 30°, 1.6 for 45°, 1.4 for 90°, 1.5 for 135°, and 1.4 for 180° were also fairly constant but showed a trend to increase for ϕ = 30° and 45°. A mixed-design ANOVA on the log-transformed thresholds with within-subject factor the value of ϕ and between-subject factor age group revealed significant main effects of the value of ϕ (*F*[1.625, 30.866] =40.05, *p* < .001) and of age group (*F*[1, 19] = 9.09, *p* = .007) but no significant interaction—*F*(1.625, 30.866) = 1.81, *p* = .186. The lack of interaction means that the trend for the ratios to increase for small values of ϕ was not significant.

**Figure 3.**
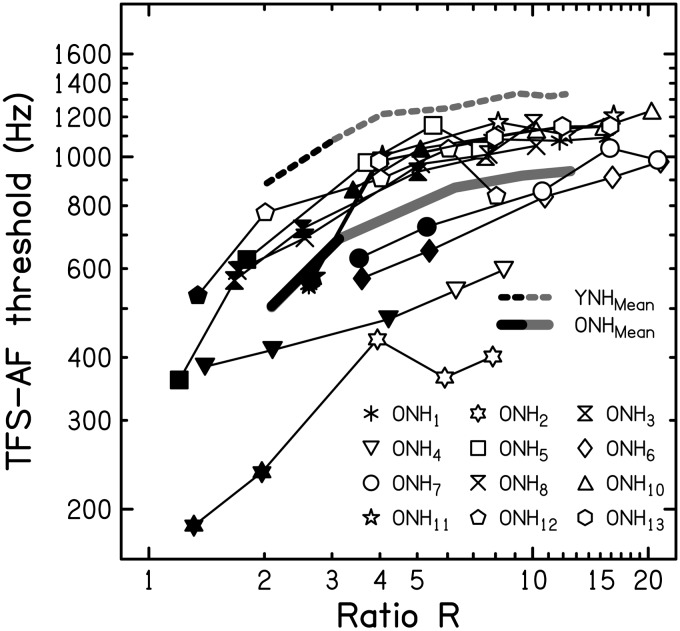
Mean individual thresholds (thin lines) and overall mean thresholds (continuous thick black and gray line) for the TFS-AF test as a function of the ratio R: ϕ expressed relative to individual and mean TFS-LF thresholds for 250-Hz tones, respectively (R expressed on a logarithmic scale). Filled symbols indicate TFS-AF thresholds that were more than 15% below that for ϕ = 180°. The thick dashed line indicates mean thresholds for nine YNH listeners from Füllgrabe et al. (2017). TFS-AF = temporal fine structure-adaptive frequency; TFS-LF =temporal fine structure-low frequency; YNH = young normal-hearing; ONH = older normal-hearing.

Figure 3 shows the TFS-AF threshold for each ONH listener plotted as a function of the value of ϕ divided by the TFS-LF threshold in degrees for a fixed frequency of 250 Hz, which is denoted R. Filled symbols indicate thresholds that were more than 15% below the threshold for that listener for ϕ = 180°. The continuous and dashed thick gray lines represent mean data for the ONH listeners and for the YNH listeners from Füllgrabe et al. (2017), respectively.

While the YNH listeners tested by Füllgrabe et al. (2017) showed very strong and significant correlations between TFS-AF thresholds for values of ϕ between 30° and 180° and TFS-LF thresholds for 250-Hz pure tones (Pearson’s *r* ranging from − .82 to −.90), the ONH listeners showed weaker correlations (Spearman’s *ρ* ranging from −.147 to −.818). For the latter, the only significant correlation was observed for the lowest value of ϕ (*p* = .001; two-tailed). Also, there was no specific value of R for ONH listeners below which thresholds declined, while YNH listeners consistently showed worse sensitivity for R ≤ 3 (see Figure 4 in Füllgrabe et al., 2017). These differences between the ONH and YNH listeners may have occurred because, for the YNH listeners, binaural TFS sensitivity appears to be a global property that characterizes a listener over a wide frequency range (Füllgrabe et al., 2017), while for ONH listeners, there may be more idiosyncratic variations in TFS sensitivity across frequency. If this were the case, then in future research studies, it might be useful to characterize sensitivity to binaural TFS using the TFS-LF test with several fixed frequencies, in addition to using the TFS-AF test. Alternatively, the weaker correlations for the ONH listeners may have occurred because the inherent variability of their threshold estimates was greater. To assess this, we calculated the ratio of thresholds for Run 3 and Run 1. The mean value of this ratio across the ONH listeners was 0.97, with an *SD* of 0.22 and a range from 0.70 to 1.34. The corresponding mean for the YNH listeners was 1.05, with an *SD* of 0.17 and a range from 0.71 to 1.31. Thus, the variability across runs was not markedly larger for the ONH than for the YNH listeners.

**Figure 4.**
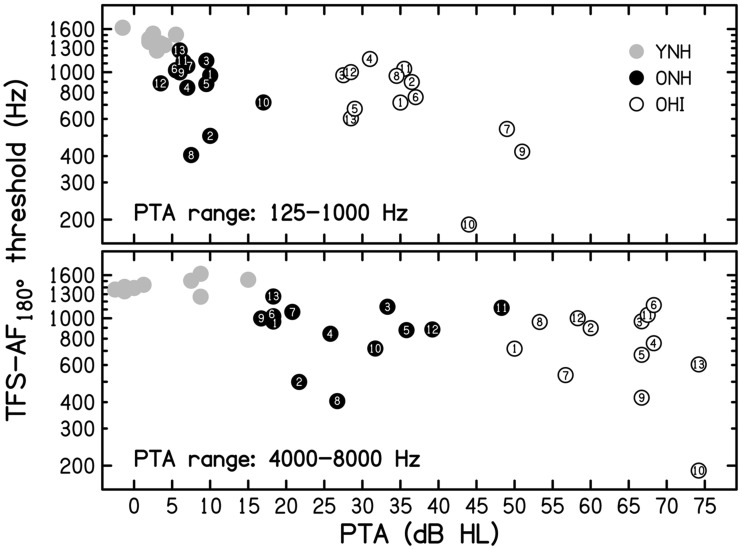
Individual thresholds averaged across runs for the TFS-AF test, using ϕ = 180°, plotted against the pure-tone average (PTA) for audiometric frequencies from 125 to 1000 Hz (top panel) or from 4000 to 8000 Hz (bottom panel) for 13 older hearing-impaired (OHI) listeners (open symbols), 13 older normal-hearing (ONH) listeners (black-filled symbols; replotted from Figure 1), and nine young normal-hearing (YNH) listeners (gray-filled symbols; replotted from Füllgrabe et al., 2017). The symbols for the ONH and OHI listeners are numbered as in Table 1, in order of increasing age. TFS-AF = temporal fine structure-adaptive frequency.

Overall, it appears that the TFS-AF test can give a graded measure of binaural TFS sensitivity for a wide range of values of ϕ and that the potential ambiguity of lateral position associated with ϕ = 180° does not have any clear adverse effects. To make the task as easy as possible, so that the listener “knows what to listen for” at the start of a run, a relatively large value of ϕ, such as 180°, seems a reasonable choice.

## Experiment 3: The Relationship of TFS-AF Thresholds to Audiometric Thresholds and Feasibility of the TFS-AF Test for OHI Listeners

### Rationale and Method

While binaural TFS sensitivity has been shown to decline with age across the adult life span (e.g., Füllgrabe et al., 2015), and this as early as midlife (Füllgrabe, 2013; Grose & Mamo, 2010; Ross et al., 2007), binaural TFS sensitivity does not seem to be related to individual differences in audiometric threshold at the test frequency when audiometric thresholds are in the normal or near-normal range and the age of the listeners is controlled for (e.g., Füllgrabe, 2013; Füllgrabe et al., 2015; Moore et al., 2012a). However, King et al. (2014) tested 46 listeners with a wide range of ages and degrees of hearing loss and found weak but significant correlations between TFS sensitivity as measured by the TFS-LF test and the audiometric threshold at the test frequency when the effect of age was partialled out (*r* = −.45 and −.42, *p* = .002 and .005 for pure tones with frequencies of 250 and 500 Hz, respectively). Moore and Sek (2016b) found a similar correlation (*r* = −.43, *p* = .05) between TFS-AF thresholds for ϕ = 180° and audiometric thresholds at 500 Hz for 22 mostly older listeners (aged 56 to 86 years) with audiometric thresholds at 500 Hz ranging from 8 to 60 dB HL (mean of 35 dB HL). They did not report whether the correlation remained significant when the effect of age was partialled out. Thorup et al. (2016) assessed binaural TFS sensitivity using an adaptive frequency-tracking task similar to that of Ross et al. (2007). For a group of 29 mostly OHI listeners (aged 52 to 80 years), they did not find a significant correlation between binaural TFS scores and audiometric thresholds averaged across the frequencies 250, 500, and 1000 Hz.

We reasoned that a clearer relationship between binaural TFS sensitivity and audiometric thresholds might become apparent by comparing results for older listeners falling into two distinct groups in terms of hearing sensitivity: normal hearing versus hearing impaired. It has also been speculated that elevated thresholds in the high-frequency range, even though not directly affecting the processing of TFS information in the low-frequency range, could act as an (early) marker of changes in TFS sensitivity (Moore et al., 2012a; Smoski & Trahiotis, 1986; Strelcyk & Dau, 2009). One aim of this experiment was to clarify the link between audiometric thresholds at both low and high frequencies and binaural TFS sensitivity.

The TFS-AF test is based on the assumption that IPD discrimination worsens with increasing frequency. However, it is possible that some HI listeners with low-frequency hearing loss are particularly insensitive to TFS cues at low frequencies, in which case, the adaptive tracking procedure would not work appropriately. As noted earlier, Moore and Sek (2016b) found that the TFS-AF test could be performed consistently by older listeners, many of whom had hearing losses at low frequencies. We wished to confirm this finding for an independent group of listeners.

To test if audiometric thresholds per se affect the processing of binaural TFS cues and if TFS sensitivity can be reliably assessed for OHI listeners using the TFS-AF test, a group of OHI listeners was tested three times on the TFS-AF test using ϕ = 180° and their results compared with those for the first three threshold runs of the YNH and ONH listeners. As before, TFS-AF thresholds were log-transformed for all inferential analyses.

### Listeners

Thirteen older listeners (five females) aged from 68 to 85 years (*M* = 77.2 years; *SD* = 6.0) were tested. Most had relatively flat mild-to-moderate low-frequency hearing losses (on average, a 4-dB decline per octave frequency between 125 and 1000 Hz) with similar audiometric thresholds for the two ears (interaural differences ≤ 15 dB). The sensorineural nature of the hearing losses was confirmed by air-bone gaps ≤ 15 dB at 500, 1000, and 2000 Hz. The lower part of Table 1 indicates listener characteristics and individual and mean audiometric thresholds for the OHI listeners.

### Results

On average, a threshold run was completed in 4 min and 58 s (excluding one outlier with a run time exceeding 9 min), a duration very similar to that for the two other groups. The dashed thick black line in the top panel of Figure 1 shows the mean results for each of the three runs for the OHI listeners. There was no trend for performance to improve across the three runs, indicating an absence of practice effects. To confirm this, and to provide a measure of the repeatability across runs, we calculated the ratio of thresholds for Run 3 and Run 1. The mean value of this ratio across the OHI listeners was 1.0, with an *SD* of 0.14 and a range from 0.67 to 1.23. The mean, *SD*, and range were similar to those for the YNH and ONH groups, as described earlier.

Figure 4 shows the individual TFS-AF thresholds (geometric mean for the first three threshold runs for each listener) for the OHI listeners (open symbols), ONH listeners (black-filled symbols; replotted from Figure 1) and YNH listeners (gray-filled symbols; replotted from Füllgrabe et al., 2017), plotted as a function of low-frequency pure-tone average (PTA) averaged across audiometric frequencies from 125 to 1000 Hz (top panel) or of the high-frequency PTA averaged across audiometric frequencies from 4000 to 8000 Hz (bottom panel). Twelve of the 13 OHI listeners gave threshold estimates clearly above the start frequency of 200 Hz. Listener OHI_10_ (aged 81 years) gave considerably lower thresholds, but all of his runs were valid (as defined earlier). His final threshold was close to 190 Hz, which is well above the lowest allowed frequency of 30 Hz.

The data points for the older listeners are numbered in order of increasing age within each experimental group in Figure 4 (see also Table 1). While there was an effect of age on binaural TFS sensitivity when comparing young to older listeners, as described earlier, TFS-AF thresholds did not decline with age within the combined group of older listeners (Spearman’s *ρ* = −.123, *p* = .550; two-tailed), perhaps because of the limited age range of these listeners.

The mean threshold was lower for the OHI than for the ONH listeners (699 and 869 Hz, respectively), but the difference was not significant according to a one-tailed Mann–Whitney test (*U* = 60.50, *p* = .109; a one-tailed test was used because we were testing the hypothesis that the TFS-AF threshold would be lower [worse] for the group with hearing loss).

To explore whether there was a gradual change in TFS-AF threshold with PTA that might not be apparent when comparing the ONH and OHI listeners, a correlational analysis was conducted for the entire group of older listeners (ONH and OHI listeners combined). Because the PTAs were not normally distributed, Spearman’s *ρ* was calculated. Using a one-tailed test (uncorrected for multiple comparisons), TFS-AF thresholds were significantly correlated with low-frequency PTA (125–1000 Hz;** Spearman’s *ρ* = −.444, *p* = .012) but not with high-frequency PTA (4000–8000 Hz; Spearman’s *ρ = *−.275, *p* = .085).

## General Discussion

The results showed that a graded measure of binaural TFS sensitivity could be obtained using the TFS-AF test for all ONH and OHI listeners, in contrast to the TFS-LF test. As for the TFS-LF test, practice effects were not apparent for the TFS-AF test. Therefore, the TFS-AF test is suitable for evaluation of binaural TFS sensitivity in the clinic or in large-scale research studies.

The effect of age found here using the TFS-AF test might reflect age-related declines in monaural or binaural TFS sensitivity, or both. Moore et al. (2012b) reported moderate correlations between scores on the TFS-LF test and scores on the TFS1 test (a monaural test of sensitivity to TFS) for NH listeners with ages from 22 to 61 years. This might indicate that part of the age-related decline in binaural TFS sensitivity reflects limitations in the processing of TFS at a stage before binaural interaction. It is also possible that the age-related decline reflects a general age-related worsening in the efficiency of auditory processing (e.g., Füllgrabe et al., 2015; Patterson, Nimmo-Smith, Weber, & Milroy, 1982). Finally, there may be an age-related increase in auditory distraction produced by task-irrelevant changes (e.g., Getzmann, Gajewski, & Falkenstein, 2013); in the present study, the frequency changes might have been distracting. However, this possibility is not borne out by a recent study showing no increase in auditory distraction with age for intramodal task-irrelevant changes (Leiva, Andrés, & Parmentier, 2015).

The ranking of performance on the TFS-AF test of YNH and ONH listeners did not vary markedly with the value of ϕ. In other words, the test could give an estimate of the relative performance of different listeners regardless of the value of ϕ. However, a large value of ϕ is recommended for routine use to ensure that the task is as easy as possible for all listeners at the start of a run, independent of their age and hearing sensitivity. This helps to indicate “what to listen for.”

As described in the Introduction section, similar tests of binaural TFS sensitivity have been used in previous studies (Grose & Mamo, 2010; Neher et al., 2011; Ross et al., 2007; Santurette & Dau, 2012; Thorup et al., 2016). However, little or no data were provided about practice effects or about the variability across runs. Grose and Mamo (2010) and Neher et al. (2011) used a presentation method that was similar to the one used for the TFS-AF test. They used 100% amplitude-modulated pure tones with four modulation cycles within each interval. In one interval, the IPD “flipped” in value between 0° and 180° in successive modulation cycles, while in the other two intervals, the IPD was always 0°. The task was to identify the interval in which the IPD flipped. Neher et al. (2011) reported a high test–retest correlation of .89, but this does not rule out the possibility that performance improved consistently in their listeners between Run 1 and Run 2. The total duration of a trial (7 s) for the method used by Neher et al. was longer than for the TFS-AF test (4.3 s). The stimuli used by Grose and Mamo (2010) were shorter (0.8 s per interval, 2.4 s for the three intervals, interstimulus interval not specified), but they used a 5-Hz modulation rate, as opposed to the 2-Hz rate used by Neher et al.; the faster rate might make the task more difficult (Blauert, 1972). As far as we are aware, the TFS-LF and TFS-AF tests are the only tests of binaural TFS sensitivity for which data on practice effects are available.

Another commonly used measure of binaural TFS sensitivity is the binaural masking level difference (BMLD; e.g., Neher, 2017; Santurette & Dau, 2012). The BMLD requires two threshold measurements (e.g., N_0_S_0_ and N_0_S_π_), although the N_0_S_π_ threshold alone has sometimes been used as an estimate of binaural TFS sensitivity. A disadvantage of the BMLD is that it depends partly on the use of energy and ENV cues (as well as TFS cues), and the cues used by HI listeners may vary depending on the severity and nature of the loss (Mao, Koch, Doherty, & Carney, 2015). Also, large training effects can occur for the N_0_S_π_ condition (Hafter & Carrier, 1970).

The TFS-AF procedure led to unrealistically high thresholds on about 3% of individual runs. In principle, the incidence of such high thresholds could be reduced by using a 3-up, 1-down procedure (tracking 79.4% correct) instead of a 2-up, 1-down procedure (tracking 70.7%) or by using three intervals instead of two, but both of these would require an increase in the total time required to achieve a fixed number of reversals.

One run of the TFS-AF test took, on average, about 5 min. We consider next how many runs would be required for 95% of the mean threshold estimates across those runs to fall within a factor of 1.3 of the “true” value (i.e., within 0.77 to 1.3 times the true value). For the log values, this corresponds to a range of ±0.114 about the mean, since log_10_(1.3) = 0.114. We based the analysis on the data for the ONH listeners because these are more representative of the population encountered in the clinic, and we assumed that the true values of the mean and the *SD* for each listener could be estimated with negligible error from the 15 estimates obtained. We denote the mean and *SD* of the log values across the 15 estimates as mean_15_ and SD_15_. The average value of SD_15_ across the 13 ONH listeners was 0.096. For a subset of *n* threshold runs, the standard error (*SE*) of the mean is ${\text{SD}}_{15}/\sqrt{n}$*.* We would expect 95% of estimates based on *n* threshold runs to fall within the range mean_15_ ±2SE. Hence, we can estimate the required number of runs as (2SD_15_/0.114)^2^ = 2.8. To the nearest whole number, this means that three runs would be needed to attain the desired degree of accuracy. This would require about 18 min including breaks, which would be somewhat too long for application in clinical practice, if each run was started “manually” by the clinician. However, it would be perfectly possible to set up a fully automated system, in which the three runs for the TFS-AF test, together with any other desired test, were run automatically with suitable breaks programmed into the software. The clinician would then merely have to collect the results at the end of testing.

We consider next the possible applications of the TFS-AF test in the clinic. If a listener shows relatively poor performance on the test, this would be expected to be associated with a reduced ability to segregate sounds based on binaural TFS information (Neher et al., 2011, 2012). Such a listener might be a candidate for hearing aids that make use of information from IPDs at low frequencies to enhance interaural level differences (ILDs), as described by Moore, Kolarik, Stone, and Lee (2016). This can lead to improved localization of speech and might improve the ability to understand speech in the presence of competing talkers, although the latter has not yet been assessed. Another possibility for listeners with poor binaural TFS sensitivity is to use binaural beamforming hearing aids, which can selectively amplify sounds from a specific direction, but at the expense of discarding IPD and ILD cues (Launer, Zakis, & Moore, 2016). The loss of IPD cues would not be a major disadvantage for a listener who already has poor sensitivity to such cues. However, a listener with good sensitivity to IPD cues might suffer more from the loss of IPD cues, so binaural beamforming might be less appropriate for such a listener (Neher, Wagener, & Latzel, 2017).

The results provided some evidence for a link between TFS-AF thresholds and audiometric thresholds at low frequencies. For the two older groups combined, the Spearman correlation between TFS-AF thresholds and audiometric thresholds averaged over the range 125 to 1000 Hz was weak to moderate (−.44). The correlation is of a similar magnitude to those reported King et al. (2014) and Moore and Sek (2016b). Thorup et al. (2016) did not find a significant correlation between binaural TFS sensitivity and audiometric thresholds at low frequencies, but the range of low-frequency audiometric thresholds in their study was relatively small, which may have limited the ability to find a correlation. Overall, it appears that there is a link between binaural TFS sensitivity and audiometric thresholds at low frequencies, but the effect of hearing loss is small relative to the effect of age, at least for hearing losses in the mild-to-moderate range.

So far, our evaluations of the TFS-AF test have focused on listeners with reasonably symmetric hearing. It would be useful in future studies to assess how well the test works for people with asymmetric losses. Neher (2017) measured BMLDs for listeners with both symmetric and asymmetric hearing loss at low frequencies and found no significant effect of asymmetry, suggesting that binaural TFS processing was still possible for the asymmetric group. If so, then we would expect the TFS-AF test also to be applicable to listeners with asymmetric hearing loss. However, this remains to be assessed.

## Conclusions

The TFS-AF test gives a graded measure of binaural sensitivity to TFS for listeners with a wide range of ages, including listeners with mild-to-moderate hearing loss. There are no effects of practice for the TFS-AF test, making the test suitable for application in the clinic or in large-scale research studies. The choice of the fixed value of the IPD does not appear to be critical, but a value of 180° is recommended to make the task easy at the start of an adaptive run.

The results confirm that binaural TFS sensitivity is worse for older than for younger listeners and provide weak support for the idea that binaural TFS sensitivity also declines with increasing low-frequency hearing loss. The effect of age seems to be greater than the effect of hearing loss. There are large individual differences in binaural TFS sensitivity for individuals with similar ages and with similar audiometric thresholds, especially among older listeners.
